# Histoplasmosis of the Larynx

**DOI:** 10.1016/S1808-8694(15)31187-3

**Published:** 2015-10-19

**Authors:** Fernando Pochini Sobrinho, Marinella Della Negra, Wladimir Queiroz, Ulisses José Ribeiro, Sérgio Bittencourt, Giselle Burlamaqui Klautau

**Affiliations:** 1 M.S student in Otolaryngology, MD. Assistant Physician of the Otolaryngology Service Hospital Nossa Senhora de Lourdes; 2 PhD. Professor, Head of the 2nd inpatient unit of the Emílio Ribas Infectology Institute; 3 M.S. in Infectology. Assistant Physician − 2nd inpatient unit of the Emílio Ribas Infectology Institute; 4 Otolaryngologist, Head of the Otolaryngology Service - Hospital Nossa Senhora de Lourdes; 5 Otolaryngologist, Head of the Otolaryngology Service - Hospital Nossa Senhora de Lourdes; 6 M.S. in Health Sciences. Assistant Physician − 2nd inpatient unit of the Emílio Ribas Infectology Institute

**Keywords:** carcinoma, granulomatosis, histoplasmosis, larynx, tuberculosis

## Abstract

The incidence of laryngeal histoplasmosis is low when compared to the total number of cases. Less than 100 cases of laryngeal histoplasmosis have been described in the medical literature. Isolated laryngeal involvement may lead to the misdiagnosis and mistreatment of tuberculosis or laryngeal cancer, according to some reports. The development of hoarseness in a patient with histoplasmosis or a laryngeal mass leading to hoarseness, might be suggestive of laryngeal histoplasmosis. When histoplasmosis is included among the differential diagnoses of a laryngeal lesion, the biopsy should be limited to a small tissue fragment, enough to perform histological tests. The laboratory technicians must be warned about the possibility of histoplasmosis, because special dyes have to be used to confirm this diagnosis.

## INTRODUCTION

Recently, granulomatous diseases, caused by infectious agents, have occurred more frequently than they were described before. Such occurrence may be explained by the increase in the number of patients with immune diseases, or patients who are submitted to treatment by immunosuppressive agents, and the growing number of elderly patients with immunosuppression.

Laryngeal histoplasmosis is a rare condition that may mimic tuberculosis or even laryngeal cancer, and must be considered in the differential diagnosis of a patient with hoarseness, diagnosed with histoplasmosis and, moreover, in the differential diagnosis of tumors in the vocal folds, causing hoarseness. Biopsy must be limited, removing the least possible amount of tissue and the pathologist must be told of the possibility of using special dyes for diagnostic confirmation.

Amphotericin B is the drug of choice for the treatment of this disease, although other drugs have also been used.

## LITERATURE REVIEW

### DIFFERENTIAL DIAGNOSIS

Histoplasmosis is a granulomatous disease of universal distribution, common, caused by a dimorphic intracellular fungus, Histoplasma capsulatum. According to Goodwin Jr. et al^1^, it was Monbreun, in 1934, who first saw that the histoplasma had two ways of growing, in room temperature (as a filamentous fungus) and in body temperature (like yeasts). The fungus invades the reticularendothelial system.

There are endemic areas, such as in the valleys of rivers Ohio and Mississipi^2^, in the USA, where about 80-90% of the population is affected.^3^

This fungus can be found in caves, hen houses or even on the ground contaminated by bats' or birds' feces.^3^ After the organisms are inhaled, there is a primary infection. The factors that impact its progression are:

1. The number of organisms inhaled;
2. The patient's age (less than 6 years or elderly citizens);
3. Immune status (immunosuppression);
4. Nutritional status (malnutrition).

Furcolow classifies histoplasmosis in3:

1) Acute pulmonary histoplasmosis
1.1. assymptomatic
1.2. symptomatic: mild, moderate and severe;
2) Chronic pulmonary histoplasmosis;
3) Acute spread histoplasmosis;
4) Cronic spread histoplasmosis:
4.1. mucocutaneous (oral cavity, pharynx, larynx, skin, lips);
4.2. meningitis;
4.3. pericarditis;
4.4. adrenal failure;
4.5. bone marrow;

There are two types of primary infection according to the number of organisms inhaled: 1. Infection with a moderate number of organisms, and 2. Infection with a massive number of organisms inhaled, usually in bat caves or in hen houses. In the former, there is asymptomatic or mild respiratory infection with one or few primary pulmonary sites. In the latter, the picture is much worse, there are multiple and simultaneous primary sites, if the disease is not treated then, it progresses to cavitation or some type of spread disease.

With the primary infection there is a fast and transitory systemic spread. For most patients the disease is limited to this point. For most immunocompetent patients, it is asymptomatic or is similar to a cold in its symptoms.^4^

In acute spread cases, usually in children below one year of age or in severely immunosuppressed patients, there is fever, weight loss, liver and spleen enlargement and pancytopenia, and the patient may develop shock, respiratory failure and disseminated intra-vascular coagulation (DIVC). In the cases when the infection extends - the acute spread, there may also be adrenalitis, endocarditis, meningitis, and ulcerations in the oral mucosa and in the intestine. In the chronic forms there is fever, weight loss, asthenia, liver and spleen enlargement and oral mucosa lesions are very common. In the acute forms, ulcerations were much less frequent.^1^

Chronic pulmonary histoplasmosis is very much similar to tuberculosis, with slow progressive fever, weight loss and respiratory symptoms with chest x-rays showing areas of excessive cavitation and fibrosis.^1^ It must especially be considered in patients who do not respond to tuberculosis treatment, in whom the tuberculosis agent was not identified.^5^ Differently from the patients with the spread form of the disease, these here do not have the involvement of other organs (muco-cutaneous, meningitis, pericarditis, adrenal failure forms of the disease). Staloff et al.^2^ commented that the spread is very rare, as a clinically apparent infection, happening more frequently in immunosuppressed and elderly patients, and more commonly in men. When the infection spreads, the histoplasma can invade almost any organ. The areas commonly involved are: bone marrow, lymph nodes, adrenal glands and gastrointestinal tract, just as the tongue and oral mucosa.

Thus, laryngeal involvement occurs in the mucocutaneous form of the chronic spread disease. Common initial manifestations are: pain to swallow, hoarseness, gingival ulceration and dysphagia.^6^ It is also common for these patients to complain of weight loss, malaise and fatigue.^5^ Hepatoesplenomegaly can occur in 30-50% of the cases, as well as skin ulcerations. Mucosa ulcerations are the most frequent findings in these patients. They may last for long periods of time in any area of the larynx. Firm, painful ulcers, with elevated borders, involving the tongue, oral mucosa and the larynx are characteristic. Submucosa masses, with or without ulcerations may also appear - mimicking carcinomas. These ulcerations can involve the oral mucosa, the gum, the tongue, lips and pharynx.^3^

About 40-75% of adults and 18% of children with progressive spread histoplasmosis have oropharyngeal involvement.^1^,^5^ The clinical meaning of these oropharyngeal lesions is primarily diagnostic. In a study involving 79 cases of histoplasmosis, Goodwin Jr. et al.^1^ reported that the mucosa ulceration distribution in the groups with acute, sub acute and spread chronic disease occurred in oropharyngeal and laryngeal involvement in 19% for the acute form, 31% for sub acute, and 66% of the cases with spread chronic diseases. They also reported that, as far as the site and frequency of such involvement is concerned for the 58 oropharyngeal and laryngeal lesions; only 7 were located in the larynx in 29 cases of spread histoplasmosis.^1^

Local involvement of oral and laryngeal mucosa can represent the only sign of spread infection.^7^,^8^ During examination, the laryngeal mucosa can be pearl-white and edematous, or inflamed and ulcerated. The lesions can be similar to carcinoma or tuberculosis. Although most authors agree that the laryngeal manifestations indicate spread lesions, there are cases reported in which the laryngeal lesions were considered the primary ones.

For Smith, Utz^9^ only 15% of the patients had isolated laryngeal lesions, however 42% manifested the oro-laryngeal disease.

According to Stallof et al.^2^, Hutchinson (1952) described one case in which the disease happened specifically on the right vocal fold.

Wihters, Pappas^10^ described a similar case, but one that did not have signs of spread.

Bhalla et al.^11^, studying the causes of acquired non-neoplastic laryngo-tracheal stenosis noticed that histoplasmosis was also part of the differential diagnosis, together with sarcoidosis, tuberculosis, osteoplastic tracheoplasty and Wegener's granuloma.

The lesions histopathologically classified by granuloma formation tend to form nodules by ulceration with elevated borders, while the lesions histopathologically characterized by histiocytic aggression (groups of parasited macrophages), associated with a more devastating disease, were superficial and more insignificant in appearance.^1^,^12^

Clinical diagnosis can be achieved with high levels of suspicion, especially in endemic areas, multiple calcifications seen in the chest x-ray, immunological tests, and the histological finding of the histoplasma in culture.^5^

One can collect a smear from the center of the ulcer for microscopy and culture. Special dyes usually show numerous macrophages with hifas, such as Gomori silver methenamine, Schiffi-Gridley periodic acid dye, with a superiority achieved with silver dyes (Grocott) when compared to PAS.^5^

The histoplasma is an intracellular organism that can be obtained from smears of ulcers in the oral cavity or from biopsies of oral and laryngeal tumors. During active infection, it is possible to see hifas in the macrophages, which are easily stained by PAS and Groccot (silver). It is also possible to see granulomas. Culture samples can come from sputum, bronchoalveolar secretions or from biopsy material. The organisms can be recovered in the Sabourauds' medium.

Microscopically, histoplasmosis lesions can have tuberculoid granulomas with caseous necrosis areas, making it difficult to differentiate it from tuberculosis.

Immune tests are: skin test with histoplasmine (in endemic areas, because of its high positiveness, its use is reduced). Staloff et al.^2^ reported that over 85% of the samples from the population of endemic areas had positive skin tests. The complement fixation tests, which, contrary to the former, are the indirect and more valuable diagnostic procedure, become positive four weeks after the infection onset. After disease resolution, titles drop and usually disappear. Complement fixation tests are fundamental in order to establish a diagnosis, as well as to assess treatment response. In practice, titles of 1:4, 1:8 indicate the disease. Titles of 1:16 or a 4 fold or higher change in the titles are highly suggestive of infection. A negative complement fixation test does not rule out the spread form.^3^

The only diagnostic certainty is achieved by isolating the microorganism.^2^ Biopsy shows granulomatous tissue, frequently necrosed, with an infiltrate of gigantic cells, lymphocytes, plasmocytic cells and a large number of macrophages. The organisms are not easily seen with the hematoxyline-eosin dye. However, with the Gomori silver methenamine, the macrophage appears with a variable number of oval and round bodies, surrounded by a clear zone. These bodies represent an intracellular hifa of the H. capsulatum.^3^

Laryngeal histoplasmosis must be treated similarly to the other forms of the disease. Although benign, there can be dissemination with severe or even fatal manifestations. Treatment is carried out with endovenous anphotericin B, 0.3-0.6 mg/kg of body weight per day, with a total dose of 2-4 mg. Mucosal lesions respond quickly (6-8 weeks), although there may be recurrencies.^3^

Goodwin Jr. et al.1 reported that a serum level of anphotericin B of 1.56 mg/mL for 10 weeks, with a total of 1g of intravenous medication, was usually enough for cure, or, when the serum dosage is not available, one can use 50mg per day or three times a week, at a total dose of 2.0g. In children, 1mg/Kg/day for six weeks has been tried. Of their 84 cases treated with amphotericin B, 11 did not cure completely and required a new treatment.

Fletcher, Prussin^7^ suggested that in immunocompetent patients with laryngeal histoplasmosis, one or two months of Ketoconazole could be administered, and if there is improvement, treatment can remain for six months.

Staloff et al.2 reported a case of histoplasmosis affecting the larynx only, treated with 200 mg of Ketoconazole tid for 3 months. The patient underwent objective tests 14 months after treatment, with good results.

Fernandez et al.^13^ reported successful treatment with itraconazole.

Negroni et al.^14^ treated 17 histoplasmosis patients with itraconazole, 100mg daily until clinical cure and, then, changed it to 50mg/day for six more months. All the infections were clinically cured, or improved greatly. 12 cases had clinical cure, 4 extreme relief and 1 patient, who interrupted treatment after 2 months of itraconazole, died.

## CLINICAL CASE

The patient came with hoarseness, progressive dysphagia, and having lost 10Kg in 2 months. He had had AIDS since 1996. At admission his blood work up showed 2 CD4 + lymphocytes per mm^3^. Direct laryngoscopy was carried out and showed a white necrotic lesion spread throughout his larynx, edema and exophytic lesion in the upper right border of the epiglottis. We biopsied the lesions. We did not observe lesions on the skin. His adrenocortical, myelogram, CSF, ecocardiogram and skull CT scan were all normal. The result was histoplasmosis. The patient was submitted to a treatment with amphotericin B, with improvement and was discharged following a treatment with fluconazole.

**Figure 1:**
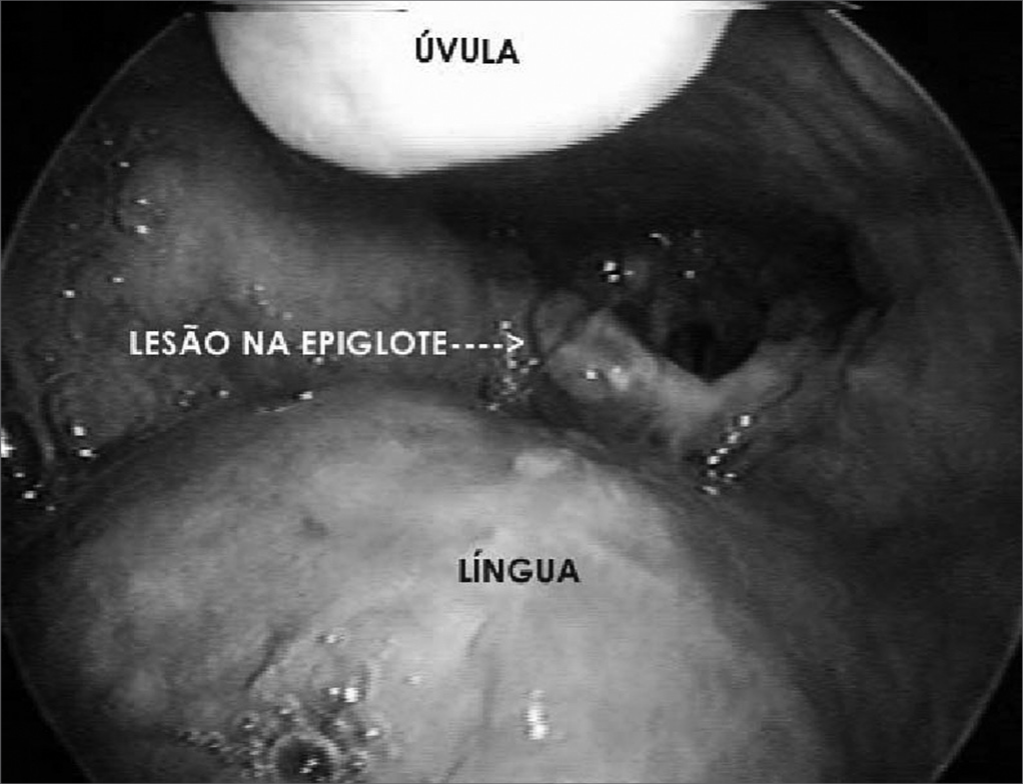
Lesion on the right upper surface of the epiglottis - laryngeal histoplasmosis.

**Figure 2:**
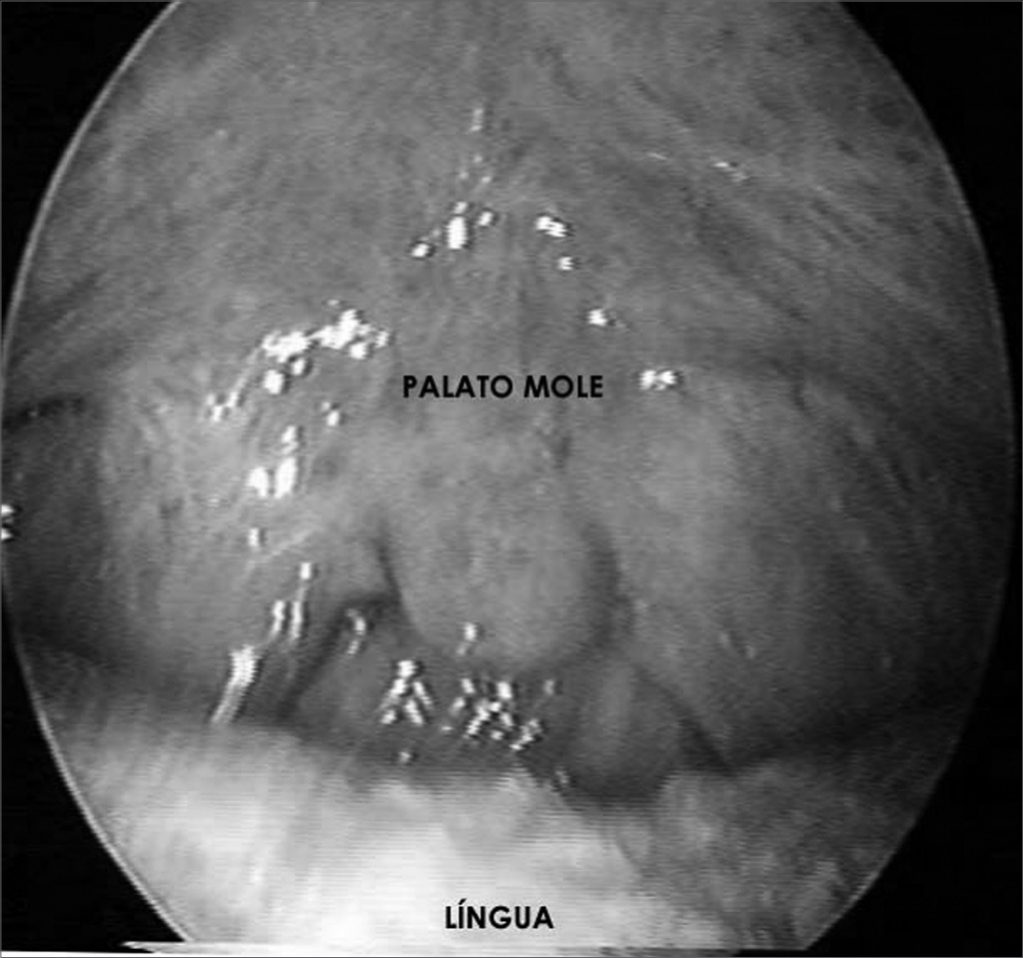
Soft palate and tongue without apparent lesions - laryngeal histoplasmosis.

## DISCUSSION

Since 1952, when laryngeal histoplasmosis was initially described in the literature, less than 100 cases have been reported^2^. These figures are small if compared to the number of patients diagnosed with histoplasmosis every year. However, we must consider laryngeal involvement in the disease if a patient with histoplasmosis develops hoarseness.^2^

Macroscopically, histoplasmosis must be differentiated from carcinoma, middle line granuloma, mucormycosis, lymphoma, syphilis, tuberculosis and other head and neck granulomatous diseases. Microscopically, it may be mistaken with:

1. paracoccidioidomycosis, with similar appearance;
2. tuberculosis in granulomatous histoplasmosis there are reports of central caseous necrosis - similar to tuberculosis;
3. squamous cells carcinoma - of atypical epithelial response.

A tumor present in the vocal fold must also be included in the differential diagnosis of vocal fold tumors causing hoarseness, as well as in those cases in which the patient does not respond to tuberculosis treatment - in which the tuberculosis bacillus was not identified. Since usually the diagnosis requires a biopsy, when it is suspected in the preoperative, the biopsy must be limited, remove the least possible amount of tissue in order to establish the diagnosis. The pathologist must also be aware of the need to use special dyes. It is recommended an accurate assessment in order to rule out spread disease.

## FINAL REMARKS

Differential diagnosis is of paramount importance, and one must consider carcinoma, mid-line granuloma, mucormycosis, lymphoma, syphilis, tuberculosis and other granulomatous diseases of the head and neck. Rajah^5^ treated, what he thought was tuberculosis in one of his patients for 10 months before the correct diagnosis, while Staloff et al.^2^ had their patient undergo two surgical procedures by another team with the diagnosis of vocal fold polyps, and later they suspected of papillomatosis. Gerber et al.^15^ carried out chemotherapy and radiotherapy to treat cancer for 2 to 3 months, without improvement and achieved good results after instating proper treatment.
